# Short-Term Outcomes of Infection-Related Glomerulonephritis in Diabetes Mellitus

**DOI:** 10.7759/cureus.67238

**Published:** 2024-08-19

**Authors:** Arjunlal T S, Thirumalvalavan Kaliaperumal, Edwin Fernando, Srinivasaprasad N D, Sujith Surendran, Poongodi Annadurai, Anila A Kurian

**Affiliations:** 1 Nephrology, Stanley Medical College, Chennai, IND; 2 Nephrology, Government Stanley Medical College and Hospital, Chennai, IND; 3 Renopathology, Renopath Center for Renal and Urological Pathology Pvt. Ltd., Chennai, IND

**Keywords:** irgn, diabetes mellitus, kidney, glomerulonephritis, infection

## Abstract

Background and objective

Infection-related glomerulonephritis (IRGN) in adults, particularly the diabetic population, has a grave prognosis with many patients progressing to dialysis-dependent renal failure. Indian data on this entity are very scarce. This study attempts to correlate the clinicopathological factors related to diabetic IRGN and its short-term outcomes.

Subjects and methods

A retrospective analysis of all diabetic patients with biopsy-proven IRGN between January 2017 and August 2021 was conducted. Factors affecting outcomes such as clinical characteristics, urine examination, complete blood count, serum biochemistry, renal biopsy, and follow-up data were obtained and analyzed to determine the risk of progression to chronic kidney disease (CKD)/end-stage renal disease (ESRD). Univariate/multivariate analysis and receiver operating characteristic (ROC) curve were performed to identify independent risk factors affecting outcomes.

Results

A total of 40 diabetic patients with IRGN was included in the study, with a mean age of 53.08 ± 10 years, comprising predominantly males (60%). Infective foci were occult in majority (37.5%). Isolated low C3 levels were documented in the majority, while three patients (7.5%) had normal complement levels. Complete renal recovery was noted in 15 patients (37.5%), while 12 patients (30%) progressed to ESRD. Anuria or uremia at presentation, glomerulosclerosis >28.6%, interstitial fibrosis with tubular atrophy (IFTA) >17.5%, and diabetic nephropathy correlated to poor renal recovery. No correlation was observed between endocapillary proliferation, the pattern of deposits, the prevalence of crescents, and complement levels with the outcome.

Conclusion

IRGN is a common immune-mediated clinical entity among diabetics and often requires renal replacement therapy. Anuria or uremia at presentation, diabetic nephropathy, elevated glomerulosclerosis, and IFTA were associated with poor renal recovery. Complement levels and crescents had no impact on the outcome.

## Introduction

Diabetes mellitus (DM) is the leading cause of chronic kidney disease (CKD) in India [1]. The epidemiology and outcomes of infection-related glomerulonephritis (IRGN), the most common and perhaps the most serious renal event with short- and long-term repercussions, are influenced largely by the geographical, sociocultural, economic, genetic, and practice patterns of a country. In adults with IRGN, significant dissimilarities have been observed between renal outcomes within the population of a confined region [1-4]. Various factors play confounding roles in renal recovery such as pre-existing comorbidities (e.g., DM, hypertension, analgesic abuse, to name a few). Globally, studies from Italy and the United States have documented an increased incidence of IRGN in the diabetic subpopulation [4,5]. Studies also have noted that diabetic patients have lower renal recovery rates following acute insult [1-3]. Since the central acute kidney injury (AKI) registry in our country is in its infancy, a scarcity of data exists on the epidemiology and outcomes of IRGN. Existing literature from our country has inherent limitations including, but not limited to, single-centre data, under-reporting, under-recognition, retrospective design, and varied definitions [6-8]. At our centre, we noted that 60% (n = 72) of patients with biopsy-proven IRGN over the past five years had DM as a comorbidity.

Though the short-term morbidity and mortality of IRGN are better recognised, a critical knowledge gap exists regarding the long-term consequences in IRGN survivors, which have clinical and public health implications. The conventional ideology of benign outcomes in IRGN survivors has been recently challenged by observational studies. It is well established that any acute insult relates to an increased risk of development of de novo CKD, or progression of existing CKD, to such extent that AKI and CKD are now recognised as mutually interconnected syndromes facilitating one another [9,10]. Additionally, in a recent single-centre study from South India, 30% of IRGN among adults progressed to end-stage renal disease (ESRD) [1]. Available regional data suggest the incidence of IRGN in diabetics with rapidly progressive renal failure was over six times that of the nondiabetic adult rapidly progressive renal failure (RPRF) population [11]. Hence, IRGN survivors represent a high-risk population imparting a significant burden on the patient and public health resources, calling for risk stratification and mitigation measures.

This study aims to analyse the etiology and short‑term (90 days) outcomes in patients with IRGN among the diabetic mellitus subpopulation in a tertiary care centre. Further, the study intends to identify the risk factors associated with CKD development or progression and mortality in these patients.

## Materials and methods

Subjects and methods

This study was a retrospective, observational study conducted in our tertiary care hospital between January 2017 and August 2021.

Patient selection and data collection

Adult patients (age ≥ 18 years) with DM and a biopsy-proven IRGN were included in the study. As no single clinical or pathological finding is pathognomic for IRGN, the diagnosis was based on the criteria put forth by Nasr et al. [12]. At least three of the following five criteria are required: (i) clinical or laboratory evidence of infection preceding or at the onset of glomerulonephritis; (ii) depressed serum complement; (iii) endocapillary proliferative and exudative glomerulonephritis; (iv) C3-dominant or co-dominant glomerular immunofluorescence staining; and (v) hump-shaped subepithelial deposits on electron microscopy (EM). Data on demographic characteristics, etiology, clinical features, comorbidities, and biochemical parameters including but not limited to urine routine examination, complete blood count, serum biochemistry, body fluid cultures, fasting, and post-prandial blood sugars, as well as C3 and C4 levels, were taken; histopathology, treatment provided, renal replacement therapy, and outcomes were retrieved from patient case records using a standardized data form. An extensive search for any active focus of infection was done in every patient with an emphasis not to miss any occult active infection. The sepsis screen included evaluation of the ear, nose, throat, and oral cavity including teeth; dermatology evaluation; echocardiography to rule out infective endocarditis; chest X-ray; and ultrasound abdomen. Laboratory parameters were measured at admission, serially, and at the discharge or before death as deemed required. Patient status was followed up for a period of up to 90 days to determine prognosis.

Outcome

The data were analysed regarding the demographic features, etiology, laboratory parameters, histopathology, and renal replacement therapy (RRT) against 90-day outcomes. The primary outcome was a composite of de novo CKD (defined by eGFR < 60 mL/min/1.73 m^2^) or CKD progression (decline in eGFR category to any higher stage) in patients with baseline CKD, at 90 days. The composite outcome of de novo CKD (defined by eGFR < 60 mL/min/1.73 m^2^) or CKD progression (decline in eGFR category to any higher stage) to ESRD warranting RRT or death at 90 days was studied as the secondary outcome.

Statistical methods

To describe the data, descriptive statistics, frequency analysis, and percentage analysis were used for categorical variables, and mean with standard deviation (SD) or median with interquartile range (IQR) were used for continuous variables. The association between qualitative variables was evaluated with the X^2^ test (chi-square) or Fisher's exact test. Quantitative variables were summarized in their mean ± SD or median and IQR. The quantitative variables were analysed using Student’s t-test (in comparisons of one variable with two categories) and/or the analysis of variance (ANOVA). Univariate and multivariable logistic analyses were used to explore the risk factors associated with in-hospital death. The risk factors with p < 0.05 for primary and secondary outcomes identified with univariate analysis using the chi-square test were further assessed using binary regression analysis. Co-linearity was analysed between the covariates. Statistical significance was considered at a p < 0.05, and an odds ratio with a 95% confidence interval was also calculated. Statistical analysis was done using IBM SPSS statistics (version 23.0) software.

## Results

Within the predefined study period, 40 patients were diagnosed to have had DM and biopsy-proven IRGN in our centre and were included in the final analysis.

Socio-demographic and clinical characteristics and etiology

The group comprised predominantly males (n = 24, 60%) rather than females (n = 16, 40%), with more female patients warranting RRT at presentation than males (Table 1). The mean age of our study population was 53.08 ± 10 years, with no significant age difference between both genders (p = 0.001). Of the patients included in the study, 12.55% had newly diagnosed DM at admission. In the remaining 35 patients, the mean duration was DM was 6.75 ± 4.07 years, with only 45.7% on regular treatment. History of nonsteroidal anti-inflammatory drug (NSAID) abuse was noted in three patients (7.5%), while smoking and alcoholism were present in 55% and 42.5%, respectively. Oliguria (87.5%) with features of volume overload (92.5%) predominated at clinical presentation, while new onset hypertension and uremia were observed in 60% and 45%, respectively. All seven patients who had anuria at presentation showed endocapillary proliferation, while crescents were evident in four patients. A total of 22 (55%) patients warranted haemodialysis at presentation.

**Table 1:** Patient clinical characteristics NSAID - Non-Steroidal Anti-Inflammatory Drugs, RRT - Renal Replacement Therapy, HIV - Human Immunodeficiency Virus, SD - Standard Deviation

Parameter		Total (n=40)	Fully Resolved (n=15)	Non-RRT Dependent (n=13)	RRT Dependent (n=12)	p value
n (%)	Male	24 (60)	9 (37.5)	7 (29.2)	8 (33.3)	0.808
	Female	16	6 (37.5)	6 (37.5)	4 (25)	
Age [years, (Mean ± SD)]		53.08 ± 10	49.07 ± 2.75	56.92 ± 3.00	53.92 ± 2.43	0.129
Comorbidities	Hypertension (n, %)	24 (60)	3 (18.8)	8 (50)	5 (31.3)	0.081
	Chronic Liver Disease (n, %)	1 (2.5)	0 (0)	1 (100)	0 (0)	-
	Diabetic Nephropathy (n, %)	9 (22.5)	0 (0)	3 (33.3)	6 (66.7)	0.008
	Hypothyroidism (n, %)	1 (2.5)	0 (0)	1 (100)	0 (0)	-
	Malignancy (n, %)	1 (2.5)	0 (0)	1 (100)	0 (0)	-
	Psoriasis (n, %)	1 (2.5)	1 (100)	0 (0)	0 (0)	-
	Pulmonary Tuberculosis (n, %)	1 (2.5)	0 (0)	1 (100)	0 (0)	-
Smoking (n, %)		22 (55)	8 (36.4)	5 (22.7)	9 (40.9)	0.183
Alcohol (n, %)		17 (42.5)	6 (35.3)	4 (23.5)	7 (41.2)	0.368
Duration of Diabetes Mellitus [years, (Mean ± SD)]		6.75 ± 4.07	5.58 ± 1.20	62.3 ± 1.25	8.85 ± 1.09	0.196
Newly detected Diabetes Mellitus (n, %)		5 (12.5)	3 (60)	2 (40)	0 (0)	0.275
Diabetes Mellitus treatment compliance (n, %)		19 (54.3)	4 (21.1)	6 (31.6)	9 (47.4)	0.123
History of Acute Kidney Injury (n, %)		0 (0)	0 (0)	0 (0)	0 (0)	-
History of NSAID abuse (n, %)		3 (7.5)	2 (66.7)	0 (0)	1 (8.3)	0.406
Newly detected hypertension (n, %)		24 (60)	12 (50)	5 (20.8)	7 (29.2)	0.081
Fever (n, %)		13 (32.5)	4 (30.8)	5 (38.5)	4 (30.8)	0.800
Duration of fever [days, (Mean ± SD)]		2.35 ± 4.15	0.86 ± 0.41	4.08 ± 1.64	2.33 ± 1.05	0.124
HIV infection (n, %)		1 (2.5)	0 (0)	1 (100)	0 (0)	0.345
Oliguria (n, %)		35 (87.5)	11 (31.4)	13 (37.1)	11 (31.4)	0.091
Duration of oliguria [days, (Mean ± SD)]		6.13 ± 4.02	5.00 ± 1.15	8.85 ± 1.04	4.50 ± 0.67	0.008
Anuria (n, %)		7 (17.5)	1 (14.3)	1 (14.3)	5 (71.4)	0.031
Duration of anuria [days, (Mean ± SD)]		2.57 ± .079	2 ± 0	2 ± 0	2.80 ± 0.37	0.568
Fluid volume overload (n, %)		37 (92.5)	13 (35.1)	13 (35.1)	11 (29.7)	0.406
Uremia (n, %)		18 (45)	2 (11.1)	7 (38.9)	9 (50)	0.004
Mean interval to presentation		7.97 ± 4.48	8.27 ± 1.51	9.62 ± 0.96	5.83 ± 0.74	0.101
Infective Foci	Diabetic foot ulcer (n, %)	14 (35)	2 (14.3)	6 (42.9)	6 (42.9)	0.346
	Skin infection (n, %)	8 (20)	5 (62.5)	2 (25)	1 (12.5)	
	Dental caries (n, %)	1 (2.5)	1 (100)	0 (0)	0 (0)	
	Lung infection (n, %)	2 (5)	0 (0)	1 (50)	1 (50)	
	Unidentified (n, %)	15 (37.5)	7 (46.7)	4 (26.7)	4 (26.7)	
Diabetic Neuropathy (n, %)		14 (35)	2 (14.3)	6 (42.9)	6 (42.9)	0.082
Diabetic Retinopathy (n, %)		26 (65)	6 (23.1)	10 (38.5)	10 (38.5)	0.035
Peripheral Vascular Disease (n, %)		10 (25)	2 (20)	6 (60)	2 (20)	0.098
Need for RRT (n, %)		22 (55)	2 (37.5)	8 (36.4)	12 (54.5)	<0.001
Mean Duration of Hospital Stay (days)		17.28 ± 8.03	12.93 ± 1.53	19.85 ± 2.95	19.92 ± 1.27	0.025

The mean interval to presentation to our centre was 7.9 ± 4.8 days from onset of symptoms. Microscopic hematuria was uniformly present, but proteinuria was variable. Diabetic foot ulcers (35%) and skin infections (20%) were identified as predominant infective triggers, while no infective foci could be identified in the majority of the study group (37.5%).

Laboratory parameters and histopathology

**Figure 1:**
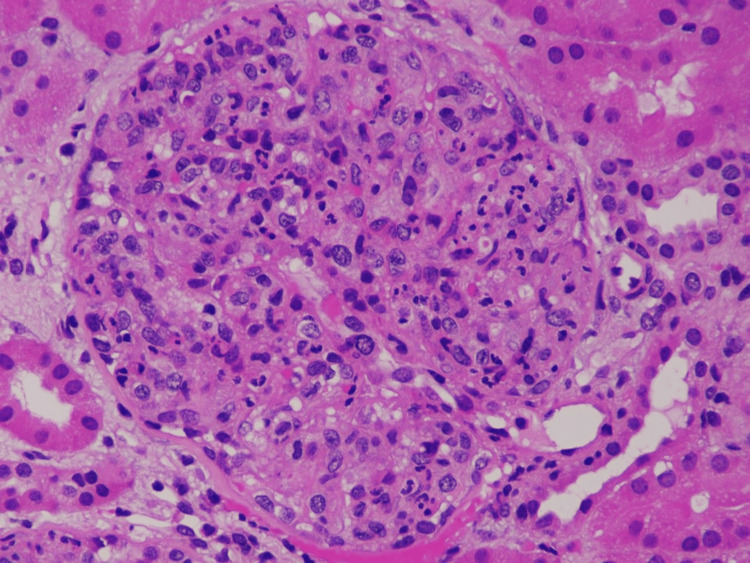
Hematoxylin and eosin staining of glomeruli showing an exudative pattern of the glomerular injury

The mean C3 and C4 complement levels were 53.6 ± 19.0 mg/dL and 19.15 ± 2.42 mg/dL, respectively, while normal complement levels were observed in three (7.5%) patients (Table 2). Endocapillary proliferation was universal, while capillary lumen obliteration was found only in one-third of cases (Figure 1). It was also noticed that a significant proportion of patients (42.5%) have had concomitant crescentic glomerulonephritis, while acute tubular injury (ATI) was identified in 24 (60%) patients. Coexisting diabetic nephropathy on histopathology was noted in nine patients, among which six patients progressed to ESRD.

**Table 2:** Patient laboratory parameters

Parameter		Total (n=40)	Fully Resolved (n=15)	Non-RRT Dependent (n=13)	RRT Dependent (n=12)	p value
Urine exam (n, %)	Blood	40 (100)	15 (37.5)	13 (32.5)	12 (30)	0.598
	Protein > 300 mg/dL	27 (67.5)	12 (44.4)	9 (33.3)	6 (22.2)	0.251
	Leucocyte esterase positive	0 (0)	0 (0)	0 (0)	0 (0)	-
	Nitrate positive	0 (0)	0 (0)	0 (0)	0 (0)	-
	RBC cast	7 (17.5)	2 (28.6)	3 (42.9)	2 (28.6)	0.792
	WBC cast	0 (0)	0 (0)	0 (0)	0 (0)	-
Nephrotic range proteinuria (n, %)		4 (10)	4 (100)	0 (0)	0 (0)	0.025
Haemoglobin (g/dL)		10.38 ±0.73	10.6 ± 0.20	10.39 ± 0.21	10.11 ± 0.18	0.214
Total leucocyte count (cells/cumm)		14632 ± 3486	12026 ± 623	15692 ± 1014	16741 ± 686	<0.001
Serum creatinine	Admission	6.28 ± 4.06	4.62 ± 1.43	7.00 ± 0.76	7.58 ± 0.66	0.125
	Discharge	3.53 ± 2.69	1.21 ± 0.41	3.05 ± 0.43	6.95 ± 0.47	-
Serum complement level (mg/dL)	C3	53.6 ± 19.0	58.73 ± 4.69	47.77 ± 4.89	53.5 ± 6.11	0.322
	C4	19.15 ± 2.42	19.2 ± 0.34	19.23 ± 0.48	19 ± 1.10	0.969
Low C3 alone (n, %)		36 (90)	14 (38.9)	12 (33.3)	10 (27.8)	0.655
Low C4 alone (n, %)		0 (0)	0 (0)	0 (0)	0 (0)	
Low C3 and C4 (n, %)		1 (2.5)	0 (0)	0 (0)	1 (100)	
Normal complement levels (n, %)		3 (7.5)	1 (33.3)	1 (33.3)	1 (33.3)	

Immunofluorescence showed predominant C3 deposits over IgG in 70%, while a codominant and IgG predominant pattern was noted only in 5% each. We also observed co-staining c1q, IgA, and IgM staining in 10%, 5%, and 5% of patients, respectively (Table 3). An IgA-dominant IRGN pattern was found in two patients, and both progressed to ESRD.

**Table 3:** Patient histopathological parameters (40 patients) RBC - Red Blood Cell, IFTA - Interstitial Fibrosis and Tubular Atrophy, RPS - Renal Pathology Society, IgG - Immunoglobin G, IgA - Immunoglobin A

Light Microscopy		Total (n=40)	Fully Resolved (n=15)	Non-RRT Dependent (n=13)	RRT Dependent (n=12)	p value
Glomeruli	Viable (%)	79.87 ± 21.66	96.16 ± 2.85	67.85 ± 7.32	54.67 ± 6.05	<0.001
	Glomerulosclerosis (%)	20.13 ± 21.66	3.83 ± 2.85	32.15 ± 7.32	45.32 ± 6.04	<0.001
	Glomerulosclerosis > 30% (n, %)	15 (37.5)	(1 (6.7)	6 (40)	8 (53.3)	0.004
	Necrotising lesion (n, %)	0 (0)	0 (0)	0 (0)	0 (0)	-
	Enlarged & hypercellular (n, %)	22 (55)	11 (50)	6 (27.3)	5 (22.7)	0.191
	Mesangial matrix expansion (n, %)	9 (22.5)	3 (33.3)	3 (33.3)	3 (33.3)	0.952
	Endocapillary proliferation (n, %)	40 (100)	15 (37.5)	13 (32.5)	12 (30)	-
	Obliteration of capillary lumen (n, %)	30 (75)	12 (36.8)	8 (34.2)	10 (28.9)	0.387
	Double contours and spikes (n, %)	2 (5)	1 (50)	0 (0)	1 (50)	0.591
	Kimmelstiel Wilson lesions (n, %)	3 (7.5)	0 (0)	1 (33.3)	2 (66.7)	0.263
	Capsular drops (n, %)	0 (0)	0 (0)	0 (0)	0 (0)	-
	Hyaline caps (n, %)	1 (2.5)	0 (0)	0 (0)	1 (100)	0.302
	Podocyte hyperplasia (n, %)	0 (0)	0 (0)	0 (0)	0 (0)	-
	Capillary microaneurysms (n, %)	1 (2.5)	0 (0)	0 (0)	1 (100)	0.302
	Crescents (n, %)	17 (42.5)	5 (29.4)	5 (9.4)	7 (41.2)	0.400
	Cellular crescents (n, %)	12 (70.6)	5 (41.7)	2 (16.7)	5 (41.7)	0.138
	Fibro-cellular crescents (n, %)	4 (23.5)	0 (0)	3 (75)	1 (25)	
	Fibrous crescents (n, %)	1 (5.9)	0 (0)	0 (0)	1 (100)	
Tubules	Acute tubular injury (n, %)	24 (60)	9 (37.5)	9 (37.5)	6 (25)	0.618
	Cytoplasmic vacuoles in tubular epithelial cells (n, %)	16 (40)	5 (31.3)	6 (37.5)	5 (31.3)	0.780
	RBC cast (n, %)	8 (20)	5 (62.5)	1 (12.5)	2 (25)	0.225
Interstitium	Interstitial edema (n, %)	27 (67.5)	10 (37)	6 (22.2)	11 (40.7)	0.052
	Nil inflammation (n, %)	29 (72.5)	15 (51.7)	11 (37.9)	3 (10.3)	0.002
	Mild inflammation (n, %)	7 (17.5)	0 (0)	1 (14.3)	6 (85.7)	
	Moderate inflammation (n, %)	4 (10%)	0 (0)	1 (25)	3 (75)	
	Severe inflammation (n, %)	0 (0)	0 (0)	1 (25)	3 (75)	
	IFTA (%)	12.75 ± 18.19	2.2 ± 1.70	15 ± 5.93	30 ± 7.79	<0.001
	IFTA > 30%	8 (20)	0 (0)	2 (25)	6 (75)	0.005
Vascular	Arterial fibro-intimal proliferation (n, %)	16 (43.2)	4 (25)	6 (37.5)	6 (37.5)	0.218
	Arteriolar hyalinosis (n, %)	17 (43.6)	1 (5.9)	7 (41.2)	9 (52.9)	<0.001
Diabetic nephropathy (n =9)	RPS I (n, %)	0 (0)	0 (0)	0 (0)	0 (0)	0.276
	RPS IIa (n, %)	1 (11.1)	1 (100)	0 (0)	0 (0)	
	RPS IIb (n, %)	7 (77.8)	2 (28.6)	0 (0)	5 (71.4)	
	RPS III (n, %)	1 (11.1)	0 (0)	0 (0)	1 (100)	
	RPS IV (n, %)	0 (0)	0 (0)	0 (0)	0 (0)	
Immunofluorescence						
Pattern of immune complex deposition	Granular over capillary loops (n, %)	28 (70)	10 (35.7)	13 (46.4)	5 (17.9)	0.100
	Granular over capillary loops and mesangium (n, %)	11 (27.5)	3 (27.3)	2 (18.2)	6 (54.5)	
	Linear over capillary loops (n, %)	1 (2.5)	0 (0)	0 (0)	1 (100)	
Immune complex staining	C3 only	2 (5)	0 (0)	0 (0)	2 (100)	0.198
	IgG more than C3	2 (5)	1 (50)	0 (0)	1 (50)	
	C3 more than IgG	32 (80)	11 (33.6)	14 (45.4)	7 (20.8)	
	Co-dominant IgG and C3 deposition	2 (5)	1 (50)	1 (50)	0 (0)	
	IgA dominant	2 (5)	0 (0)	0 (0)	2 (100)	

Outcome

Of 40 patients included in the study, haemodialysis was employed as an RRT modality in 22 (55%). At 90 days, only 15 patients (37.5%) had complete renal recovery defined as GFR > 90 mL/min/1.73m^2^ and absence of proteinuria (Table 4). Of the remaining 62.5% of patients, 13 (32.5%) patients progressed to CKD and another 12 (30%) patients culminated in ESRD on RRT, respectively. We did not observe any mortality during the study period.

**Table 4:** 90-day outcomes of the study population LR - Likelihood Ratio, NSAID - Non-steroidal Anti-Inflammatory Drugs, # - Endocapillary Proliferation is a constant in both groups.

90-Day Outcomes	Total [n (%)]	
N	40 (100)	
Lost to follow up	0 (0)	
n (after excluding loss to follow up)	40 (100)	
Fully recovered	15 (37.5)	
Primary outcome (de novo CKD (eGFR < 60 mL/min/1.73 m^2^) or CKD progression (decline in eGFR category to any higher stage) in patients with baseline CKD)	25 (62.5%)	
Mortality	0 (0)	
Secondary outcome (de novo CKD (eGFR < 60 mL/min/1.73 m^2^) or CKD progression (decline in eGFR category to any higher stage) in patients with baseline CKD or death)	25 (62.5%)	
RRT dependency	12 (30)	
Factors influencing RRT dependency at 90 days	LR	p value
Fever	0.005	0.941
Oliguria	0.291	0.590
Anuria	6.388	0.011
Fluid overload	0.017	0.897
Uremia	6.390	0.011
Male gender	0.322	0.571
Diabetic nephropathy	6.950	0.008
Diabetic retinopathy	2.739	0.098
Diabetic neuropathy	1.657	0.198
RRT warranted at admission	11.553	<0.001
Peripheral vascular disease	0.670	0.413
Crescents	1.749	0.186
Arteriolar hyalinosis	9.489	0.002
Interstitial edema	5.320	0.021
Acute tubular injury	0.707	0.400
Endocapillary proliferation	#	#
Obliteration of capillary lumen	0.670	0.413
History of hypertension	0.020	0.888
NSAID abuse	0.017	0.897
Alcohol	1.749	0.186
Smoking	2.882	0.090
New-onset hypertension	0.020	0.888
Newly detected DM	3.865	0.049
DM drug incompliance	3.274	0.070
Low complement levels	0.017	0.897

Risk factors associated with primary and secondary outcomes

**Figure 2:**
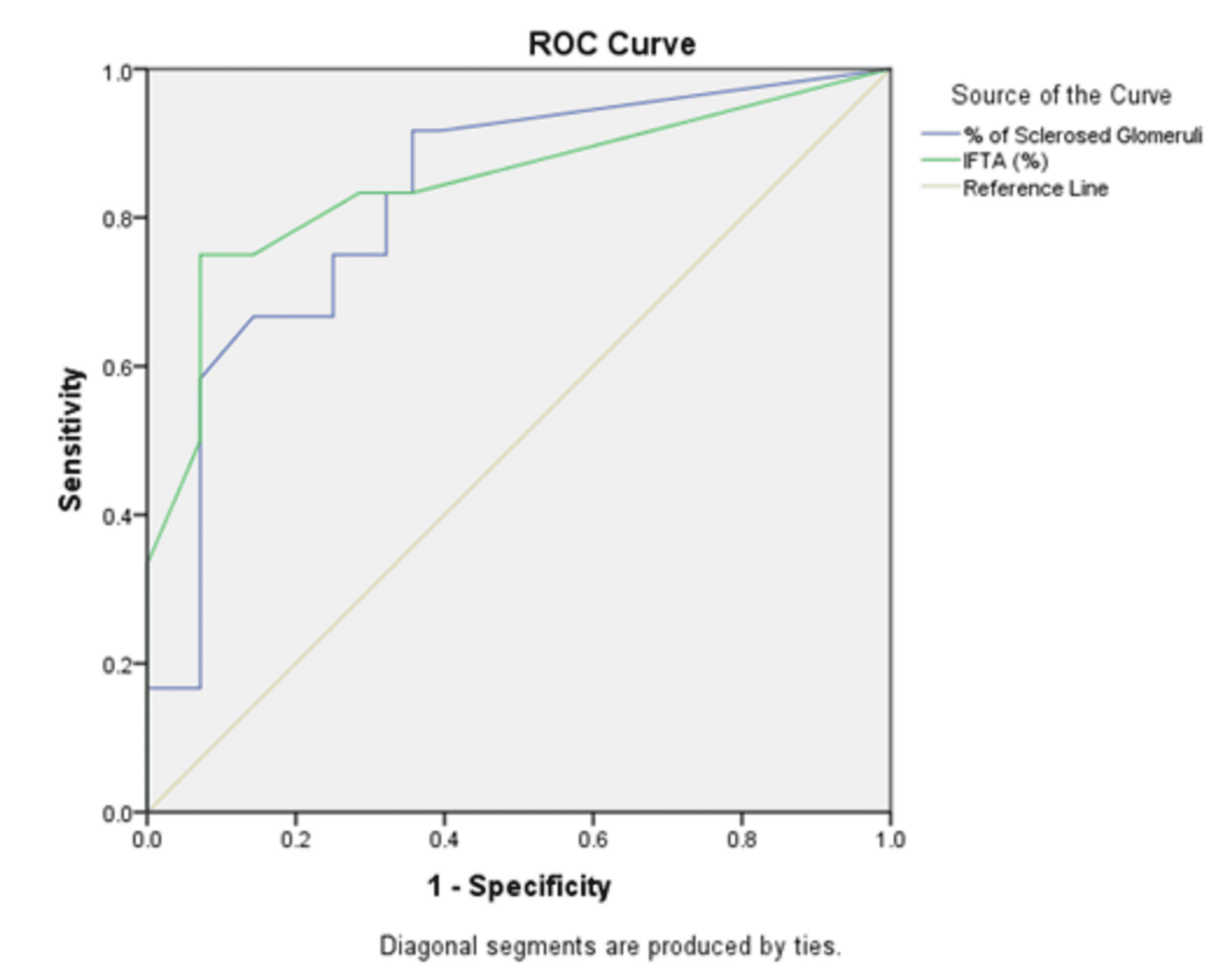
ROC curve depicting glomerulosclerosis and IFTA on microscopy showing a positive correlation with incomplete renal recovery (Spearman’s rho = 0.001) ROC - Receiver Operating Characteristic; IFTA - Interstitial Fibrosis and Tubular Atrophy

In multivariate analysis anuria or uremia at presentation, coexisting diabetic retinopathy, intense inflammatory infiltration of interstitium, interstitial edema, arterial hyalinosis, coexisting IgG deposition over tubular basement membrane, and intensity IgG deposition (> 2+) over capillaries were observed more frequently in those with poor renal outcomes. Both glomerulosclerosis and IFTA on microscopy showed a positive correlation with incomplete renal recovery (Spearman’s rho = 0.001). Receiver operating characteristic (ROC) analysis of glomerulosclerosis > 28.6% (sensitivity of 66.7% and specificity of 82.1%) and IFTA > 17.5% (sensitivity of 75% and specificity of 92.9%) in biopsy revealed a correlation with poor renal recovery and progressing to CKD/ESRD (Figure 2). However, we did not observe any relation between endocapillary proliferation causing obliteration of the lumen, the pattern of immunoglobulin deposit, prevalence/type crescents, or complement levels with the final renal outcome.

## Discussion

Accumulating evidence on the adverse long-term consequences of IRGN in diabetics has changed our perception of this disease, generally considered to have a benign outcome. The development and validation of histopathological and immunohistochemical staining patterns, apart from use in epidemiologic studies and trials, has increased our understanding of the short- and long-term outcomes of IRGN.

More than half of our study population were males. However, we noted that, among the 22 (55%) who warranted haemodialysis upon presentation, the majority were females (odds ratio: 2.14). At our tertiary care centre, we noticed delayed presentation of patients despite one week or more since disease onset, reasons being delayed recognition, inadequate management of the precipitating cause, difficulty in health care access, resorting to alternate medicine, and delayed referral including in-hospital nephrology referral. The mean age of 53.08 ± 10 years and the mean diabetic duration of 6.75 ± 4.07 years were comparable to most of the similar studies, though some studies have reported considerably lesser and greater mean age than ours [12-15]. A significant proportion of patients had undiagnosed DM at admission and have been excluded while calculating the average duration of the said illness. Regardless, a longer duration of DM was found to correlate with poor renal outcomes in our study population. Compared to data from developed countries, IRGN is approximately 10 times more prevalent in the Indian population [1,13,15]. Males were represented more than females (1.5:1) in our study, as has been observed in other studies, which may be related to more healthcare access for males [6,16].

Infectious foci triggering the inflammatory cascade could not be identified in the majority of the cases (37.5%). In those for whom evident focus was identified, diabetic foot ulcer was the leading cause (35%), followed by cutaneous infections (20%) viz. cellulitis, boils, lower respiratory tract infections (5%), and dental caries (25%). This pattern is attributed to the wider prevalence of DM in the elderly Indian subpopulation [17,18]. Among the different sites, skin and soft tissue was the most common sepsis source in our cohort, which could have been largely prevented if they had been managed appropriately early. In a recent study by Priyamvada et al., skin and soft tissue was the most common foci, whereas other studies have cited the urogenital system, lung, or abdomen as the predominant site [1,19,20].

In our current study, 55% of patients needed renal replacement therapy, which was preferably provided as intermittent haemodialysis in the majority, while most other related studies showed a slightly lower requirement of renal replacement therapy [1,21]. At 90 days, only 15 patients showed complete renal recovery, with the remaining 25 (62.5%) patients progressing to CKD. This was significantly higher than other studies on IRGN in the general population [1,2]. This finding may be partially explained by co-existing diabetic kidney disease as evidenced by biopsy in nine patients, though their baseline GFR prior to the current renal insult was unknown. However, a total of six patients with coexisting diabetic nephropathy progressed to ESRD and continued to be on RRT at the end of 90 days. Sixteen patients (40%) with IRGN without baseline CKD developed de novo CKD at 90 days, with six patients culminating in ESRD warranting RRT. Experimental models have shown maladaptive repair or disordered regeneration or both, due to renin-angiotensin activation, tubular G2/M arrest, inflammation, epigenetic changes, and mitochondrial dysfunction among others, culminating in vascular drop-out and resulting in glomerulosclerosis and interstitial fibrosis with tubular atrophy, each of which contributes to progressive renal dysfunction by perpetuating injury and hampering repair [9,22]. AKI progresses to CKD through at least two distinct pathways, either non-recovering AKI progressing to CKD being the most established trajectory or after an ‘apparent’ recovery following AKI; a serial decline of normal renal function is hastened, which is being frequently noted in recent times [23]. In our study, we have assessed only the first pathway of non-recovering AKI progressing to CKD. Another caveat with apparent ‘complete’ recovery is that it resorts to creatinine level as a primary marker of renal recovery, which is drastically confounded by factors, such as muscle mass loss, changes in volume of distribution, and hyperfiltration [9,24]. The race is already afoot in search of biomarkers to identify ongoing renal injury, which may help in risk-stratifying patients for intervention [9,22]. We observed very low mortality in our 90-day study period, which may be partially influenced by the exclusion of patients with haemodynamic instability from the study population as they were unfit to undergo renal biopsy mandated in the inclusion criteria. In view of the scarcity of data regarding the same, no patients received oral or IV corticosteroids during their hospital stay. Regardless, in general, all studies quoted here have not documented any serious mortality risk.

Various studies across the globe show AKI to CKD progression in 8-54% of patients with IRGN [8,25-29]. In a similar study from the same region, Arivazhagan et al.'s prognosis of 45 patients with IRGN among adults in the general population was studied, and they noted that 33.3% progressed to ESRD at the end of 90 days [1]. The predominant risk factors for poor outcomes were identified to be age > 40 years, alcohol intake, peak creatinine > 1.5 mg/dL, a requirement for dialysis at presentation, and the presence of moderate-to-severe interstitial fibrosis with tubular atrophy (IFTA). These results are concurrent with our study outcomes as well, except for the higher ERSD rates in our study. Our study population comprised predominantly elderly population (age > 45 years) and coexisting diabetic nephropathy 22.5% cases may be the reason that we noted higher CKD rates.

On analysing the risk factors associated with 90-day outcomes, advanced age, anuria or uremia at presentation, diabetic retinopathy, baseline diabetic kidney disease (DKD), need for RRT, intense inflammatory infiltration of interstitium, interstitial edema alongside features of chronicity such as arterial hyalinosis, and coexisting IgG deposition over the tubular basement membrane were observed more frequently in those with poor renal outcomes. Light microscopy revealed glomerulosclerosis > 28.6% (sensitivity of 66.7% and specificity of 82.1%) and IFTA > 17.5% (sensitivity of 75% and specificity of 92.9%) in biopsy revealed a correlation with de novo CKD or progressing to CKD/ESRD. We did not observe any correlation between alcohol intake, smoking, immunoglobulin deposition pattern, serum complement levels, and crescentic transformation to renal outcome. IgA-dominant IRGN has been already established to have poor outcomes [29], which were noted in two patients in our study. The sample size was not sufficient to make any relevant remarks on the impact of NSAID abuse on renal recovery.

Despite accumulating evidence on poor long-term outcomes of IRGN among adults, barriers and knowledge gaps exist about interventions to improve outcomes. Considering the increasing incidence of IRGN in diabetics and its progression to ESRD, care provided during the admission warrants revamping to include a follow-up period, which is amenable to interventions to forestall the development of CKD and or to secure early permanent vascular access for RRT, which is often difficult in the DM patients. In the Western world, apart from a Brazilian study that showed 8% ESRD risk in IRGN, most other studies on IRGN were primarily based on children, and hence even in developed countries, follow-up care is lacking [26-29]. Another study from East India also concluded that IRGN should no longer be considered a benign disease considering its higher risk of progression to ESRD [29]. Post-IRGN risk stratification, improved processes of care including regular monitoring of blood pressure, glycemic control, proteinuria and renal function, and medication reconciliation are strategies to improve long-term outcomes for these patients down the lane.

The primary strength of our study is that we focused on diabetic patients as our study population and that we conducted an extensive search for occult infectious triggers and looked into the impact of histopathological aspects on biopsy. Our study did have a few limitations, including retrospective study design, single-centre study, small sample size, exclusion of hemodynamically unstable patients or those without renal biopsy, being a tertiary care centre, and a study population that may not be representative of the general population.

## Conclusions

The survival after IRGN in the DM subpopulation is now increasingly regarded as being a portent of adverse long-term events. Knowledge of the epidemiology and outcomes are essential to frame policies to overcome the barriers and care gaps to improve outcomes. The present study has thrown light on the epidemiological factors for IRGN and the impact of diabetes on recovery in the adult diabetes population. The severity of IRGN at presentation as marked by anuria or uremia at presentation, need for RRT, alongside other features of severe renal insult such as intense inflammatory infiltration of interstitium, interstitial edema, arterial hyalinosis, moderate-to-severe glomerulosclerosis or IFTA, and additional presence of coexisting diabetic retinopathy or nephropathy were as red flags in the development or progression of CKD in our study, while classical markers of active inflammation such as serum complement levels and presence crescents have shown to be less impactful. A longer follow-up of ‘apparently’ recovered patients is warranted to better delineate the impact of IRGN in the diabetic population.
